# Examining the Contextual Factors Influencing Intersectoral Action for the SDGs: Insights From Canadian Federal Policy Leaders

**DOI:** 10.34172/ijhpm.8108

**Published:** 2024-07-14

**Authors:** Joslyn Trowbridge, Julia Y. Tan, Sameera Hussain, Erica Di Ruggiero

**Affiliations:** ^1^Dalla Lana School of Public Health, Social and Behavioural Health Sciences Division, University of Toronto, Toronto, ON, Canada.; ^2^School of Epidemiology and Public Health, Faculty of Medicine, University of Ottawa, Ottawa, ON, Canada.; ^3^Social and Behavioural Health Sciences Divsion, Dalla Lana School of Public Health, University of Toronto, Toronto, ON, Canada.; ^4^Institute of Health Policy, Management and Evaluation, Dalla Lana School of Public Health, University of Toronto, Toronto, ON, Canada.; ^5^Centre for Global Health, Dalla Lana School of Public Health, University of Toronto, Toronto, ON, Canada.

**Keywords:** Intersectoral Action, Sustainable Development Goals, Government, Policy, Leadership, Governance

## Abstract

**Background:**

The interdependent and intersecting nature of the Sustainable Development Goals (SDGs) require collaboration across government sectors, and it is likely that departments with few past interactions will find themselves engaged in joint missions on SDG projects. Intersectoral action (IA) is becoming a common framework for different sectors to work together. Understanding the factors in the environment external to policy teams enacting IA is crucial for making progress on the SDGs.

**Methods:**

Interviews [n=17] with senior public servants leading SDG work in nine departments in the federal government of Canada were conducted to elicit information about issues affecting how departments engage in IA for the SDGs. Transcripts were coded based on a set of factors identified in a background review of 20 documents related to Canada’s progress on SDGs. Iterative group thematic analysis by the authors illuminated a set of domestic and global contextual factors affecting IA processes for the SDGs.

**Results:**

The mechanisms for successful IA were identified as facilitative governance, leadership by a central coordinating office, supportive staff, flexible and clear reporting structures, adequate resources, and targeted skills development focused on collaboration and cross-sector learning. Factors that affect IA positively include alignment of the SDG agenda with domestic and global political priorities, and the co-occurrence of social issues such as Indigenous rights and gender equity that raise awareness of and support for related SDGs. Factors that affect IA negatively include competing conceptual frameworks for approaching shared priorities, lack of capacity for "big picture" thinking among bureaucratic staff, and global disruptions that shift national priorities away from the SDGs.

**Conclusion:**

IA is becoming a normal way of working on problems that cross otherwise separate government accountabilities. The success of these collaborations can be impacted by contextual factors beyond any one department’s control.

## Background

**Key Messages**

**Implications for policy makers**

- Intersectoral action (IA) is a useful organizing approach for governments implementing actions on the Sustainable Development Goals (SDGs).
- Designing successful and sustainable IA requires attention to governance and leadership across departments and in departmental central offices.
- Policy-makers can capitalize on current social issues and global attention on the SDGs to raise awareness and support for SDG collaboration, and can align domestic policy priorities to increase public and bureaucratic support for the SDGs.
- Policy-makers may need to consider the disruptions that climate, health, or political events can have on the progress of IA due to diverting resources and attention away from the SDGs.

**Implications for the public**

The Sustainable Development Goals (SDGs) are a global agreement to end extreme poverty, protect the health of humans, animals, and the planet, and tackle global inequality. Countries are creating and implementing strategies to achieve the goals, which require government departments to work together with partners they may not have worked with previously. This study shows that there are important considerations for the success of these collaborations, such as strong leadership and matching the work the government is already doing with similar priorities for the SDGs. It also shows that things beyond a government’s control, like the COVID-19 pandemic, can impact their work on the SDGs. Better progress on the SDGs could be made if government departments were aware of these positive and negative influences on collaborative projects.

Motivating diverse government actors to collaborate on an ambitious global agenda—the 2030 Sustainable Development Goals (SDGs)—is a complex endeavour. With 17 Goals aimed at ending extreme poverty, protecting the health of the planet, and tackling global inequities, all governments must create a comprehensive strategy, implement actions, regularly monitor, and evaluate progress. The interdependent and intersecting nature of the SDGs require collaboration across government sectors. It is likely that departments with little to no previous interaction will find themselves engaged in joint missions to advance aspects of the SDGs. Institutional mechanisms that centralize this type of work vary across governments, yet the SDGs are predicated on the need for different sectors to work together for common goals.^1,2^ A common approach for working together on the SDGs is the practice of “intersectoral action,” (IA) which refers to the process by which actors from different policy areas work together to achieve a common goal that actors in a single policy area could not achieve on their own.^3^ Understanding the practice of IA is key to determining how governments can progress on the SDGs as an interconnected national and global agenda.

 The IA approach stems from global public health discourse in the 1970s, as experts grappled with the impact of non-health factors on population health outcomes. The concept was codified through World Health Organization (WHO) conferences and declarations, becoming a technical approach and statement of principle.^4^ As the field developed a broader understanding of the social determinants of health, IA became a cornerstone principle of public health, aimed at engaging disparate research and policy fields to address the underlying causes of ill health. The IA discussion in public health maps a similar conversation in the field of public administration and policy sciences. This conversation sought to advance concepts and practices of policy coherence and horizontal or multisectoral governance. The public administration literature focused on mechanisms of coordination, distribution, and institutionalization of culture, values, and power in government, as well as the effects of these issues on collaborative efforts.^5,6^

 The IA literature chronicles case studies of collaborative action between government sectors, illuminating facilitative and inhibiting factors in different sociopolitical contexts. More recent studies focus on the use of IA for progressing the SDGs, but few uncover the different factors that may impact IA’s success from the viewpoint of the policy actors engaged in collaboration on SDGs.^7^ Our study engages policy leaders in discussing the use of IA for the SDGs, focusing on the contextual factors that influence IA practices. Our research questions asked: (1) what are the contextual factors (global, social, political, economic, or other) that influence the progress of collaboration on the SDGs in Canada, and (2) how does the SDG agenda influence IA across federal departments? This paper presents the results from this investigation into the contextual factors influencing the Canadian federal government’s approach to IA for the SDG agenda.

###  Canada’s Approach to the Sustainable Development Goals

 Canada signed on to the 2030 Agenda for SDGs in September 2015 at the General Assembly of the United Nations. Canada’s first step was to conduct an audit to provide a baseline for measuring the federal government’s progress in implementing the 2030 Agenda. Between 2015 and 2017, the Office of the Auditor General conducted an audit of seven departments responsible for leading the 2030 Agenda: Employment and Social Development Canada, Environment and Climate Change Canada, Global Affairs Canada, Indigenous and Northern Affairs Canada, Status of Women Canada, the Privy Council Office, and Statistics Canada.^8^ It found that a cohesive national strategy was lacking and the federal governance structure was not optimal to facilitate cross-department collaboration. It also found that and no plans for broad public and stakeholder consultation had been made. Soon after the auditor general report’s release, the national statistics office was tasked with creating a system to measure, monitor, and report on progress, and a centralized SDG unit was created to map the 17 goals to current federal departmental priorities and mandates. Canada reported on its initial steps in the 2018 Voluntary National Review for the United Nations, committing to multi-stakeholder partnerships, but did not set out a clear governance structure for bringing diverse departments together for collaborative action.^9^

 Since 2018, the SDG Unit has launched a national engagement process and published a series of SDG action plans. The most recent is the 2021 Federal Implementation Plan, which set out national targets and measuring indicators for 30 actions across the SDGs.^10^ While it did not fully describe a national governance structure, the Federal Implementation Plan mapped out which departments would be responsible for each of the SDGs. It detailed “vertical leads” (departments responsible for work on a specific SDG), and “horizontal leads” (departments with additional responsibility for cross-cutting objectives such as gender equality and poverty reduction).

 Little is published in the academic and grey literature on the process and mechanisms by which departments in Canada create action plans for their “vertical” or “horizontal” responsibilities. The connections between departments working on similar actions, similar goals, and horizontal, cross-cutting issues is also opaque. While the SDG Unit’s reports claim IA as a key mechanism for progress, there is a research gap in how IA is working in practice. Below, we present our results from a document analysis and interviews with federal policy actors working on the SDGs. We uncover the national and global contextual factors that influence the success of IA processes across departments engaged in SDG action plans.

## Methods

###  Research Design

 This study combined a document review with semi-structured interviews of federal public servants working on SDG projects in a variety of federal departments and agencies. The document review aimed to (*a*) gather background knowledge about Canada’s plan for achieving the SDGs, (*b*) assess any progress on achieving the targets as measured by indicators and as evaluated by authors external to the government, (*c*) identify planned or in-progress collaborative efforts between departments and sectors, and (*d*) illuminate federal government priorities that relate to the SDGs. This review helped the authors situate their knowledge on the history, progress, and design (governing structures, decision-making and reporting processes) of the federal government’s approach to the SDGs and to develop questions for interviews. The key informant interviews aimed to gather perspectives on the status and progress of planned SDG collaborative activities from senior public servants working directly on SDG-related projects. The interviews solicited perspectives about the structure of project operations, such as the leading department and division, the collaborative efforts planned or in progress with other departments (including governing tables and intersectoral committees), how progress was measured, and the nature of interactions of project leaders with the central SDG Unit situated in Economic and Social Development Canada. The interviews also focused on eliciting key informants’ views of IA, the contextual factors that informants identified as impacting IA, and the challenges they faced in implementing IA in their SDG projects. Author 4 led obtained the funding and led the conception and design of the study. Authors 1 and 2 led the recruitment and interview process. Author 1 led the deidentification of interview data, coding, and initial analysis. All authors (1-4) participated in iterative group sessions for further analysis and interpretation of data. Author 1 drafted the manuscript, authors 2, 3, and 4 provided critical revision of the manuscript. Authors 1 and 2 provided administrative and technical support and author 4 provided supervision for the study.

###  Document Review

 We included four types of documents in our review (See Table 1 for the list of 20 included documents):

1. Documents published by the federal government specific to the planning and progress of the SDGs.
2. Strategies and reports published by the federal government related to sustainability, gender equity, and health equity.
3. Ministerial mandate letters issued by the Prime Minister of Canada that detail the priorities and objectives for each minister to accomplish, as well as the 2020 Speech from the Throne and economic statement.
4. Evaluations of Canada’s SDG progress and United Nations Department of Economic and Social Affairs (UN DESA) SDG reports.

**Table 1:** Documents Reviewed by Type

**Document Type**	**Included Documents**
Documents published by the federal government specific to Canada’s SDG progress	1. Office of the Auditor General of Canada. 2018 Spring Reports of the Commissioner of the Environment and Sustainable Development to the Parliament of Canada. Independent Auditor’s Report. *Report 2 - Canada’s Preparedness to Implement the United Nations’ Sustainable Development Goals.* 2018.
	2. Global Affairs Canada. *Canada’s Implementation of the 2030 Agenda for Sustainable Development. Voluntary National Review.* 2018.
	3. Sustainable Development Goals Unit, Employment and Social Development Canada. *Towards Canada’s 2030 Agenda National Strategy Interim Document*. 2019.
	4. Sustainable Development Goals Unit, Employment and Social Development Canada. *Canada’s 2030 Agenda National Strategy – Moving Forward Together.* 2021.
	5. Sustainable Development Goals Unit, Employment and Social Development Canada. *Canada’s Federal Implementation Plan for the 2030 Agenda.* 2021.
	6. Statistics Canada. *Canadian Indicator Framework for the Sustainable Development Goals Data Hub*. https://sdgcif-data-canada-oddcic-donnee.github.io/. Updated September 29, 2023. Accessed May 12, 2021.
Strategies and reports published by the federal government on topics related to the SDGs	7. Environment and Climate Change Canada. *Achieving a Sustainable Future. A Federal Sustainable Development Strategy for Canada 2019 to 2022.* 2019.
	8. Global Affairs Canada. *Canada’s Feminist International Assistance Policy. #HerVoiceHerChoice*. 2017.
	9. Public Health Agency of Canada. *From Risk to Resilience: An Equity Approach to COVID-19. The Chief Public Health Officer of Canada’s Report on the State of Public Health in Canada 2020.* 2020.
Federal ministerial mandate letters and economic statements	10. Federal Mandate Letters – Mandate letters outline the objectives each minister will work to accomplish. There were 36 mandate letters issued on December 13, 2019 by the Prime Minister of Canada, and 36 supplementary update letters issued on January 15, 2021 to reflect new priorities identified in the Speech from the Throne 2020 and the Fall Economic Statement 2020.
	11. Governor General of Canada*. A stronger and more resilient Canada: Speech from the Throne to Open the Second Session of the Forty-third Parliament of Canada.* September 23, 2020.
	12. Government of Canada. *Supporting Canadians and Fighting COVID-19: Fall Economic Statement 2020*. November 30, 2020.
Evaluations of Canada’s SDG progress and UN DESA reports	13. British Columbia Council for International Cooperation. *Where Canada Stands: A Sustainable Development Goals Progress Report Vol. I.* 2017.
	14. British Columbia Council for International Cooperation. *Where Canada Stands: A Sustainable Development Goals Shadow Report Vol. II.* 2018.
	15. British Columbia Council for International Cooperation. *Where Canada Stands: A Sustainable Development Goals Progress Report Vol. III.* 2019.
	16. Organization for Economic Cooperation and Development. *Measuring Distance to the SDG Targets 2019: An Assessment of Where OECD Countries Stand. Canada.* 2019. https://www.oecd-ilibrary.org/sites/64495bfd-en/index.html?itemId=/content/component/64495bfd-en. Accessed March 20, 2021.
	17. McArthur JW, Rasmussen K. Classifying Sustainable Development Goal trajectories: A country-level methodology for identifying which issues and people are getting left behind. *World Development.* 2019;123:104608.
	18. Sachs J, Schmidt-Traub G, Kroll C, Lafortune G, Fuller G. SDG Index and Dashboards Report 2018. Canada Dashboard. *Bertelsmann Stiftung and Sustainable Development Solutions Network (SDSN). *New York; 2018.
	19. United Nations Department of Economic and Social Affairs. *Compendium of National Institutional Arrangements for implementing the 2030 Agenda for Sustainable Development: The 46 countries that presented voluntary national reviews at the high-level political forum in 2018. Canada*. 2019: 28-31.
	20. United Nations Department of Economic and Social Affairs. *Multi-stakeholder engagement in 2030 Agenda implementation: A review of Voluntary National Review Reports (2016-2019).* 2019.

Abbreviations: SDG, Sustainable Development Goal; UN DESA, United Nations Department of Economic and Social Affairs; OECD, Organisation for Economic Co-operation and Development.

 These documents were selected based on their relevancy to Canada’s planning and progress on the SDGs between 2015 and early 2021, their potential to signify relevant policy and economic priorities of the ruling government, and their potential to identify planned or actual collaborative mechanisms that encourage IA across federal departments.

Box 1 lists the information we extracted from these documents to inform our knowledge of Canada’s SDG process. Information we looked for included directives or plans for SDG activities, indicator development and measurement, the identification of lead departments and plans for cooperation, collaboration, or interaction between departments, and issues named as federal government priorities that relate to SDG areas. We used this knowledge to develop the background section of this paper and to inform our data collection strategy for key informant interviews. Documents that listed lead departments, divisions, or units for SDG activities informed our search for key informants, and knowledge of the types of SDG activities and evaluations of their progress informed the development of our semi-structured interview guide.

**Box 1. Information Areas Extracted from Document Review for Background Knowledge**

- Name and number of SDG
- Indicator measurement of goal and sub-goals
- Progress towards goal (statistical indicators and written evaluations)
- Lead department responsible for goal
- Sectors and/or departments mentioned for collaborative efforts
- Description of in-progress or planned collaboration or IA (directly related to an SDG and indirectly related as a named federal priority action)

---------------------

Abbreviations: IA, intersectoral action; SDG, Sustainable Development Goal.

###  Key Informant Interviews

 Interviews elicited information about the activities that departments were engaged in on SDG projects, and the contextual factors affecting collaboration and IA. The semi-structured interview guide used for these conversations is included in Supplementary file 1.

####  Selection and Recruitment

 This study aimed to recruit bureaucratic staff working at a variety of senior levels (senior policy advisor, manager, assistant director, director, director general, and assistant deputy minister) across key federal departments and agencies leading SDG work. Authors 1 and 2 generated a list of departments involved in SDG activities from the document review and reviewed departmental websites to identify potential key informants and their positions. In most cases, the names of employees were not listed. We used additional search strategies including the Government Electronic Directory Services and snowball sampling to identify potential key informants and their contact information. In total, 82 key informants were identified, and contact information was found for 71 of them. Each of the 71 identified informants was invited to a one-hour online interview via a dedicated email address set up for the study. Follow-up to unanswered invitations were sent twice, for a maximum of three email interactions. The recruitment and interview period of June to August 2021 coincided with summer vacations and a snap election called on August 15, 2021, leading to many informants declining to participate or respond to the invitation. As authors 1 and 2 completed interviews, we looked for diversity or repetition in key themes related to IA, mainly the facilitators, barriers, and additional contextual factors that impact its success. As diversity narrowed and repetition occurred, we were confident we had gathered enough information-rich interview data that additional interviews would not be needed. We employed Malterud and colleagues’ concept of “information power” to guide our decision on interview saturation.^11^ They suggest that the more information a sample holds that is relevant for the study, the lower number of participants is needed. By targeting senior levels of public servants, we were able to gather detailed information from long-term policy leaders that had the capacity to reflect on the topic of IA in complex federal policy projects.

####  Interviews

 Authors 1 and 2 interviewed a total of 17 federal public servants involved in SDG projects across the following nine departments (listed alphabetically), with one informant speaking independent of department:

1. Crown-Indigenous Relations and Northern Affairs Canada
2. Employment and Social Development Canada
3. Environment and Climate Change Canada
4. Global Affairs Canada
5. Indigenous Services Canada
6. International Development Research Centre
7. Public Health Agency of Canada
8. Statistics Canada
9. Women and Gender Equality Canada
10. Independent (one informant spoke independent of department)

The job titles and seniority of key informants were:

1. Senior Policy Analyst/Advisor
2. Manager
3. Director
4. Director General/Executive Director

Interviews were conducted over Zoom between June and August 2021 by authors 1 and 2, averaging one hour in length and audio-recorded for transcription purposes. The audio recordings were transcribed by a third-party company and de-identified by authors 1 and 2 to remove identifying information (names, job titles, and employment relationships). To maintain the confidentiality and anonymity of interview participants, we confirmed that we would not list any names, job titles, or departments they were associated with in the dissemination of results. In this paper, quotes are attributed to an anonymized participant list.

####  Analysis

 Author 1 coded transcripts in NVivo software (version 12) based on a set of codes established from our interview guide and document review. As coding of transcripts progressed, we identified additional codes to represent themes emerging from the data. Table 2 presents the final coding framework. We engaged in an iterative analysis process once coding was complete. First, author 1 performed a preliminary analysis on each code across all interviews, creating a word document that presented descriptive information and identified potential themes and supporting key informant quotes. Next, we extracted key themes discussed in the word document to an excel spreadsheet to establish overarching themes, supporting sub-themes, and key points for each code. Finally, our full research team held two group theming sessions using the online collaboration whiteboard software Miro. These sessions allowed us to create colour-coded thematic groups of our key results, discuss their supporting data, and come to agreement on final themes and sub-themes. This paper details the results of these collaborative theming sessions rather than presenting the descriptive analysis of data gathered under each code.

**Table 2:** Coding Framework for Interview Data

**Code Number and Title**	**Description**
1a. SDG – Description of Activities	The department’s mandate, roles, responsibilities, and activities related to the SDGs Can include history of learning/involvement with the SDGs, and origin of mandate
1b. SDG – Governance	Any institutional structures, both formal and informal, that the department is involved with committees, connection to the SDG unit, reporting structures, training materials, etc
1c. SDG – Data Collection	Indicators, measurement, data collection, reporting to Statistics Canada Canadian Indicator Framework, Global Indicator Framework
2a. Intersectoral Action – Description of Activities	The activities the department leads in terms of collaboration or partnership with different departments/sectors Can include informants’ understanding of the concept of IA
2b. Intersectoral Action – Success Factors	Factors noted as aiding the success of IA
2c. Intersectoral Action – Challenges	Factors noted as interfering with the success of IA
3a. Contextual Factors – Politics, Platform, Party	Issues related to the political process, political environment, party platforms and priorities and their impacts on IA and SDG progress
3b. Contextual Factors – Economy and Public Funds	Issues related to the economy, federal budget, and use of public funds and their impacts on IA and SDG progress
3c. Contextual Factors – Global Factors	Issues related to global discussions of SDG progress
3d. Contextual Factors – Health Factors	Issues related to public health, healthcare, and health equity and their impacts on IA and SDG progress
3e. Contextual Factors – Social & Cultural	Issues related to social and cultural movements and discussions and their impacts on IA and SDG progress
3f. Contextual Factors – Environmental	Issues related to the environment and climate change and their impacts on IA and SDG progress
4. COVID-19 Effect on Work	The current and potential future effects on the department’s work on SDGs and IA Can include potential effects of pandemic recovery planning may have on SDG agenda
5. Policy Coherence	Information related to the systems or plans to ensure integration of SDG activities with established or future domestic policies between and across government sectors
6. Other Frameworks	Other sustainability or equity frameworks or policies that informants identify as potentially competing with the SDGs for attention and resources
7. Future Steps	Information related to future activities for SDGs and IA

Abbreviations: IA, intersectoral action; SDG, Sustainable Development Goal.

## Results

 Our aim was to uncover the contextual factors that impact IA for the SDGs in both the internal and external environment in which the action takes place. Our results illustrate some of the anticipated factors for success or failure that can be found in the IA literature.^12,13^ Our analysis also reveals new contextual influences, such as the disruption of IA due to fluctuating political mandates. Overall, we heard that implementing Canada’s SDG Agenda is complex and involves multiple federal actors. The Federal Implementation Plan requires all federal departments and agencies to integrate the 2030 agenda into their work, regardless of their individual areas of responsibility. The goals and principles of the 2030 Agenda, and the targets in the Canadian Indicator Framework, should be considered in new policy development, and existing policies and programs should align with corresponding SDG obligations. The Implementation Plan establishes “vertical” lead departments, which coordinate with other departments on a particular SDG, and “horizontal” lead departments (which may also be acting as vertical leads) that ensure the integration of crosscutting objectives across the government (gender equality, diversity, and inclusion, advancing reconciliation with Indigenous peoples, and coherence between domestic and international actions on the SDGs). Working towards common objectives and managing instances of conflicts between departments requires a commitment to successful IA and a collective aim to achieve policy coherence. The following factors impacting IA for the SDGs emerged as themes from our key informant interviews.

###  Contextual Factors That Impact IA for the SDGs Within Government

####  Facilitative Governance

 Clear governance (ie, the structures, mechanisms and processes that facilitate interactions and collaborative action) of the SDG agenda is critical to its implementation across federal departments. It can be enabled by a history of collaboration. It can also facilitate a shared vision and alignment across departments.

####  a. Enabling Governance Mechanisms

 The creation of the SDG Unit enabled *facilitative leadership*, whereby the Unit’s leaders became facilitators of planning and benchmarking inside and across diverse federal departments. The Unit created interdepartmental committees that aided IA by providing a platform for knowledge-sharing and coordination. Interview participants found the central coordinating function of the Unit as integral to helping actors see the value in cross-departmental collaboration. Aids such as planning and reporting templates, clear chains of contact, and regular networking helped operationalize departmental SDG action plans and accountability mechanisms. One participant noted that the *“multiple points and opportunities to provide input…and collaborative workshop-type meetings and bilateral discussion with colleagues in other departments…were important for success”* (Key Informant 10, Department 4).

####  b. History of Collaboration

 A history of collaboration and close ties between departments allowed some policy leaders to capitalize on their relationships and mutual understanding of priorities. Prior to the establishment of the SDG Unit as a coordinating function, some departments had already built a shared willingness to work together. For example, interview participants noted ongoing collaborative work on the Federal Sustainable Development Strategy, the Feminist International Assistance Policy, and the Poverty Reduction Strategy. They reported that the more these historical relationships were formalized, such as through Assistant Deputy Minister roundtables, the more likely IA on the SDGs would become a common and shared goal. One participant noted that some collaborative processes *“were quite well entrenched and systematized” *(Key Informant 13, Department 3)in these policy areas, and another reported that these procedural structures *“are very robust, very well entrenched” *(Key Informant 8, Department 6).Drawing on these structures and historical relationships between departments helped *“bolster current priorities” (*Key Informant 2, Department 7) related to SDG work.

####  c. Shared Vision and Alignment Across Departmental Mandates

 A governance process that created a shared vision for understanding the SDGs was found to be important for successful IA. The SDG Unit created an iterative process for learning and talking about the interconnected nature of the SDGs, which helped departmental leads explain the goals and value of the SDG agenda in their home departments. The SDG Unit helped departments with the “how-to” of creating internal assessments of current work mapped to SDG actions and led a communications campaign to raise the profile of Canada’s commitments to the SDGs. One participant stated that there exists *“more of an understanding of what the [SDG] agenda is among public servants from different departments, and there is more of a desire to align communication and a clear idea of what the [federal government] is doing to advance SDGs…we are hoping that will lead to more policy integration” *(Key Informant 17, Department 5).

 Aligning new SDG responsibilities with existing departmental mandates allowed policy leaders to capitalize on their subject-matter expertise and leadership reputations to engage other departments that were not previously connected to a particular SDG priority area. Senior-level support for SDG actions that dovetailed ministerial priorities drove the primacy of the action on the policy agenda and enabled resource support. This made it easier for teams to align their current work to an SDG action plan and to engage other sectors with more authority. As one participant noted, the alignment between priorities meant that *“you didn’t have to do too much convincing with other departments” *(Key Informant 2, Department 7). In the case of abrupt shifts in political priorities, however, SDG plans could face disruption. For example, one participant explained that as the COVID-19 pandemic grew, *“there was a shift in gears…we had been moving in one direction and then the Minister’s office said to accelerate the work in [another area], so there was a bit of pivot to doing more of a higher-level strategy [for that area]” *(Key Informant 10, Department 4). Participants also noted that an awareness of what other government levels are doing is required to ensure IA is effective. One participant noted that “*when it comes to de-centralized authority…what is required is that the [federal government] has to organize provinces [and municipalities] …and what is expected is a whole of society effort, everybody has to buy in and contribute” *(Key Informant 17, Department 5).

####  Public Sector Capacity for Intersectoral Action 

 IA for the SDGs requires significant capacity including the need to invest in and mobilize human and financial resources, and alignment of funding and reporting structures that enable IA.

####  a. Mobilizing Human Resources

 Human resources inside each department are needed to plan and lead SDG-relevant actions and to coordinate with other departments and the SDG Unit. The SDG Unit leads a citizen consultation strategy and a broad departmental engagement plan, bringing a wealth of viewpoints together to inform initial action plans. Participants felt that time, money, and training were resources required for successful IA. One participant noted that *“relationship-building, trust-building, all those things take time…they’re not just an activity you can check off the list” *(Key Informant 7, Department 8).The challenge to help departments and teams understand where they fit in the SDG framework, and how they may be sharing or overlapping priorities with other departments, is a large time investment at the outset of the SDG work. It can interfere with the day-to-day work of the department and can deter some departments from pursuing IA.

####  b. Training for Intersectoral Action

 Shifting from siloed thinking about departmental mandates to systems thinking for collective action on SDGs requires targeted training for people involved in IA. Prior to Canada signing on to the SDGs, some departments had little interaction and engagement with other sectors. Policy teams may not have had experience working with actors outside their department, or with managing change and thinking through big-picture goals. Participants discussed the *“different thought process to be able to look a little more broadly at what you’re trying to accomplish” *(Key Informant 5, Department 5). They worried that this way of thinking, as well as *“specific skills like negotiation, diplomacy, empathy, willingness to allow someone else’s agenda to go before your own” *(Key Informant 7, Department 8) were minimized due to fast-paced project planning and a lack of understanding about what training would be helpful for new IA. Without this type of training, SDG projects were at risk of being less collaborative. One participant felt that there was a clear *“institutional hesitation around stepping into what might not feel like your lane or your core responsibilities” *(Key Informant 13, Department 3).Training and skills development for IA is a core component of increasing capacity for collaboration.

####  c. Aligning Funding and Reporting Structures

 Rigid funding and reporting structures can discourage collaboration. Participants discussed the overwhelming reporting requirements to their own departments, to the SDG Unit and to broader committees. They noted that “joined-up funding” where two departments, or multiple teams inside a department, could jointly fund an SDG project, was difficult to find. One participant noted the challenge of connecting with *“different sectors, different players, that have different organizational cultures” *(Key Informant 6, Department 1). Aligning formal reporting processes and lines of accountability may help align diverse organizational or administrative cultures and allow policy actors from different sectors to work better together.

###  Contextual Factors That Influence IA for the SDGs in the External Environment

####  Global Prominence of the SDG Framework

 Factors external to the federal government’s SDG planning and implementation processes can influence the success of IA. In particular, the global prominence of the SDGs can elevate the legitimacy and urgency of IA across government departments that had been focused on internal priorities. The continuous reporting requirements to high-level UN platforms also drives departments to engage in intersectoral collaboration as they work to complete projects and measure progress on the Goals. Interview participants discussed the benefits of adopting the global SDG agenda as a method of framing their current priorities and taking bureaucratic ownership for achieving SDG benchmarks. In addition to facilitating team engagement, a participant noted that *“it gives us more strength behind our ask [and] more authority when we can also say our work is part of the SDG framework” *(Key Informant 2, Department 7). Another participant commented that the SDG framework is *“a common language that is providing a collective frame of reference” *(Key Informant 5, Department 5).However, participants talked about the potential for tension between the SDG framework, which centres health and gender equity, poverty reduction, and environmental sustainability as guiding concepts across the goals, and the numerous federal frameworks or overarching departmental strategy documents that use the same concepts. They were concerned that the primacy of the SDG agenda could be reduced if policy teams are confused by the operationalization of the SDG agenda for department projects already underway. One participant relayed that for them, *“the challenge is people wrapping their heads around: how does this [SDG agenda] fit in with that other framework or strategy? It’s more about people realizing, oh, this is slightly different, it has the word ‘sustainable’ in it but it’s not quite the same thing [as other sustainability or equity frameworks]” *(Key Informant 13, Department 3). This concern points to the need for clarity in the ways that different departments operationalize the global SDG agenda when engaging in IA.

####  Catalytic Events and Social Movements Heighten Awareness for IA and SDGs

 Participants suggested that sociostructural and environmental issues, such as the Black Lives Matter movement and major climate events, are heightening public awareness of and support for the complex issues that the SDGs tackle. They also suggested that there is a greater awareness of socio-economic gaps within Canada and globally due to the differential effects of the pandemic on some populations. This translated to a small extent to an increased awareness inside departmental policy teams working on the SDGs, and has helped drive, according to key informants, a better understanding of issue interdependence and the need to work intersectorally. For example, Canadian media had been reporting on the Black Lives Matter movement, the finding of several unmarked graves on land previously used for residential schools for Indigenous children, and the higher rates of COVID-19 in racialized communities. Speaking of these sociostructural issues, a participant reflected that, *“Certainly, the awareness of socio-economic gaps and the historical context and legacy of past policy on Indigenous peoples is giving us a bit more opportunity to leverage the SDGs to push for closing those gaps” *(Key Informant 10, Department 4). Similarly, another participant said that given the *“[current] conversation around diversity and inclusion, there’s lots of ways we have entry points into the [SDG] conversation with our stakeholders” *(Key Informant 2, Department 7).

####  a. COVID-19 Pandemic as a Positive and Negative Disruptor

 The COVID-19 pandemic disrupted most departments’ SDG action plans. Many financial and human resources were shifted from SDG work towards the pandemic response – one participant noted that *“the pandemic is going to drive a whole set of fiscal issues that are going to be enormous downstream…that fiscal context will become the narrative for the next five to ten years…and trade-offs are going to be hard for something like the SDGs” *(Key Informant 13, Department 3). However, the pandemic was also seen as a positive disruptor to the “usual way of work.” Participants felt it created a greater appreciation for systems thinking, interdependence and collaboration, which may create enabling conditions for successful IA. It also heightened awareness of persisting health, racial, and gender inequities across Canada and globally, which may allow for greater understanding of the value of the SDGs. One participant reflected that, *“COVID-19 brought us this understanding that we have to look at things differently, we need to find new ways to build back better, so there is an appetite for quality-of-life type of frameworks such as the SDGs” *(Key Informant 17, Department 5). Another participant argued that COVID-19 has shown that “*we need to think about fundamental shifts in the way in which we structure government at higher levels…it has helped recentre certain issues with the SDGs being one of the answers” *(Key Informant 1, Department 1). The full impact of the pandemic on the culture of work in government, as well as on the activities to progress the SDGs, is still unknown. Participants were worried that the pandemic diverted significant resources away from SDG progress but were hopeful that greater awareness by the public and policy-makers of the significance of poverty and inequality, displayed during the pandemic, could increase support for future government action on the SDGs.

## Discussion

 This study confirmed that IA is operationalized as an important method for achieving the SDGs in the Canadian federal government. The senior public servants we spoke to agreed IA is the best approach for making progress on the SDGs but noted that it was not formally put into use until the creation of the central task force (the SDG Unit in the Employment and Social Development Canada department). While IA has a long history of use as an approach in the health sector in Canada, it had not been applied at an overarching federal policy level until Canada adopted the SDGs in 2015.^14-17^ As IA becomes a regular approach across the federal government for SDG work, a set of contextual factors that influence the use and success of IA need attention.

 Key informants revealed the mechanisms and conditions for successful IA – central leadership, supportive staff, flexible but clear governance and reporting structures, the presence of trust, adequate resources, targeted training, and skills development. These conditions also appear in other case studies and reviews of IA processes.^12,13,18^ What is surprising is the potential for IA processes to be influenced by a variety of domestic and global contextual factors. Our findings indicate that IA is positively influenced in situations where these contextual factors elevate the SDG agenda or align political priorities, and that IA is negatively influenced when priorities and resources are misaligned, or when global events disrupt government’s “business as usual.”

 Domestic contextual factors that spur IA on the SDGs include ensuring a legislative mandate for departments to adopt the SDGs, and the alignment of individual departmental mandates to their assigned SDG priorities. When the SDG Unit created a Federal Implementation Plan that assigned SDGs to specific departments for implementation, it triggered the conditions for a governance and accountability process to get the work underway. Research on the governance of intersectoral collaborations finds that the ‘internal legitimacy’ necessary for collaboration is derived from formal leadership support for collaboration.^19^ The Federal Implementation Plan created the condition for this formal leadership support. The departments that found IA on the SDGs easier to undertake were those that already had mandates to work on policy areas like the Goal they were subsequently tasked with in the Implementation Plan. In addition, we found that a national policy agenda that aligns with the SDGs (for example, gender equity priorities) help IA processes, because it signals the importance of these overarching policy priorities. Research on IA in health policy that pre-dates the SDGs made a similar assertion that “a clear mandate and a supportive policy environment are equally desirable in fostering a sense of solidarity, facilitating collective action, [and] acknowledging the requirement for long-term investment in IA.”^14^

 Global contextual factors that elevate IA on the SDGs include the prominence of the SDG agenda itself in global forums and media, and social or political movements that highlighted or aligned with aspects of the SDGs. Our key informants felt that there was an increased awareness of the content and importance of the SDGs across departments when Canada participated in UN-level processes such as High-Level Political Forums. Similarly, some participants noted social and environmental movements as opportunities for highlighting important issues that the SDGs target, such as poverty, the climate crisis, and inequities. These global forces provide entry points for internal and external stakeholder engagement on support for, and implementation of, IA for the SDGs.

 Conversely, if political priorities for federal departments are misaligned with SDGs, or political system ‘shocks’ happen, such as elections and changes of government, IA is disrupted. Navigating collaborative action on the types of long-term issues the SDGs target is difficult if government commitments are short-term and driven by electoral cycle considerations. Major global events, such as a pandemic or a climate emergency, can also disrupt policy leaders’ commitment to IA on the SDGs as resources and mandates are re-directed towards new problems. Our analysis of key informants’ views of the COVID-19 pandemic shows that such external ‘shocks’ interrupt SDG work and can divert resources and collaborative effort away from IA. However, as the pandemic highlighted the disproportionate health and economic inequalities across communities, more people became aware of the inequity embedded in our health and social systems. According to our key informants, this awareness helped increase support for the SDGs among other public servants and external stakeholders. Recent research finds a similar mixed effect of the pandemic – it has markedly diverted resources and delayed progress on the overall SDG agenda but has created focused effort on key aspects of the agenda, such as poverty reduction and health and gender equity.^20,21^ Our findings support calls to apply a political lens to IA for health and climate problems.^22,23^ Competing political priorities, different goals for political leaders in power, and shifting social awareness of inequality are important factors shaping the success or failure of implementing IA for the SDGs.

###  Limitations

 Our interviews were conducted during the COVID-19 pandemic and immediately before a federal election. We solicited input from representatives from nine departments, but we recognize that participation may have been limited due to the pandemic impacting the personal and professional lives of senior public servants and the election diverting them from new or additional tasks such as participation in research. Our interview guide was robust and informed by our document and literature analyses, and analysis of interview transcripts revealed saturation for this study’s research questions, but we acknowledge we may have missed capturing additional views on IA and the SDGs from more departments.

## Conclusions

 Our study raises important considerations for governments invested in an IA approach to the SDGs, and for implementation of IA broadly. One, there is a risk that as IA becomes normative and required across different areas of government, processes could become fragmented and cross-purposed. Policy actors warned that adding layers of collaboration in the form of meetings, interdepartmental committees, and extra planning and reporting could overwhelm teams and stall progress. When other collaborations on health equity, gender equity, or sustainability, for example, are underway in tandem with SDG collaborations, care should be taken to ensure clear governance procedures are followed. Two, there is an opportunity to enable broad support for IA beyond the SDGs by mobilizing global platforms for agenda-setting. Social issues, such as the Black Lives Matter movement, that engage governments and publics globally can galvanize collaboration and mobilize policy actors to make connections with stakeholders outside of their routine. Finally, our study revealed that while IA is becoming a normal way of approaching complex policy priorities such as the SDG Agenda, policy leaders want more evaluation and evidence of the benefits of IA processes. As IA is increasingly operationalized and evaluated, this study suggests that attention to the contextual factors that can positively and negatively influence IA is warranted.

## Ethical issues

 This study was approved by the University of Toronto Research Ethics Board, Protocol #4083.

## Competing interests

 Authors declare that they have no competing interests.

## Supplementary files


Supplementary file 1. Semi-structured Interview Guide.
