# Supplementary material for: Examining the Contextual Factors Influencing Intersectoral Action for the SDGs: Insights From Canadian Federal Policy Leaders
Source: Int J Health Policy Manag. 2024 Jul 14;13:8108. doi: 10.34172/ijhpm.8108 (PMC11365081; doi:10.34172/ijhpm.8108)
Supplement: Supplementary file 1 — Semi-structured Interview Guide. [file ijhpm-13-8108-s001.pdf]

Article title: Examining the Contextual Factors Influencing Intersectoral Action for the SDGs: Insights From Canadian Federal Policy Leaders

Journal name: International Journal of Health Policy and Management (IJHPM)

Authors' information: Joslyn Trowbridge[1]*, Julia Y. Tan[1], Sameera Hussain[2], Erica Di Ruggiero[3,4,5]*

[1]Dalla Lana School of Public Health, Social and Behavioural Health Sciences Division, University of Toronto, Toronto, ON, Canada.

[2]School of Epidemiology and Public Health, Faculty of Medicine, University of Ottawa, Ottawa, ON, Canada.

[3]Social and Behavioural Health Sciences Divsion, Dalla Lana School of Public Health, University of Toronto, Toronto, ON, Canada.

[4]Institute of Health Policy, Management and Evaluation, Dalla Lana School of Public Health, University of Toronto, Toronto, ON, Canada.

[5]Centre for Global Health, Dalla Lana School of Public Health, University of Toronto, Toronto, ON, Canada

***Correspondence to:** Joslyn Trowbridge; Email: Joslyn.trowbridge@mail.utoronto.ca & Erica Di Ruggiero; Email: Email: e.diruggiero@utoronto.ca

Citation: Trowbridge J, Tan JY, Hussain S, Di Ruggiero E. Examining the contextual factors influencing intersectoral action for the SDGs: insights from Canadian federal policy leaders. Int J Health Policy Manag. 2024;13:8108. doi:10.34172/ijhpm.8108

Supplementary file 1. Semi-structured Interview Guide

Background	1. How did you come to learn about the SDGs? (probe for influences from other countries, other departments, research community, etc.)<br>2. Which goal(s) and targets are driving your department/unit/office's activities?<br>3. Can you tell me about your role and involvement with SDGx?<br>4. How did your office/department/ministry gain responsibility for the mandate of SDGx?
Progress & Actions	5. What are the main actions you are undertaking related to SDGx?<br>a. Probe for history of actions, types of actions, funding mechanisms<br>6. How do your actions align with the Towards Canada 2030 framework?

	7. What is helping actions on SDGx to get and stay on the federal policy agenda?<br>a. What might be hindering actions on SDGx to get and stay on the federal policy agenda?<br>8. How are you measuring progress on SDGx?<br>a. Probe Statistics Canada Data Hub, Canadian Indicator Framework, department own data collection<br>9. Does intersectoral action for the SDGs differ from intersectoral action for the social determinants of health?
Coordinating Structures, Governance Mechanisms, Feedback and Evaluation	10. Can you describe the coordination structure of actions on SDGx?<br>11. Probe for leadership, research, implementation, technical operations, budget, monitoring and evaluation roles, coordinating structures, meeting processes<br>12. How is progress measured and reported within/to these structures?<br>13. What collaboration mechanisms are in place to work across federal departments on the SDGs?<br>14. What does successful collaboration across federal departments look like to you?

Intersectoral Action	15. What does intersectoral action mean to you?<br>a. Probe other language – horizontal collaboration, partnerships, health in all policies<br>16. Can you describe the intersectoral action/collaboration/partnerships you engage in on SDGx?<br>17. What is the motivation for intersectoral action/collaboration in this work?<br>18. Is there a mandate for collaboration across federal government departments on the SDGs?<br>19. The Towards Canada 2030 framework speaks about policy coherence as an important part of Canada's approach, how does this inform your collaborations?<br>20. Are there any contextual factors that affect your approach to collaboration?<br>21. How have the pandemic and recovery plans influenced your work in this area?<br>22. What are the next steps and future actions?
Facilitators/Barriers for Intersectoral Action	23. Are there specific challenges to intersectoral action?<br>24. How are these challenges identified?
